# ^68^Ga-Trivehexin PET/CT: a promising novel tracer for primary hyperparathyroidism

**DOI:** 10.1007/s00259-024-06846-z

**Published:** 2024-07-19

**Authors:** Serkan Kuyumcu, Dilara Denizmen, Duygu Has-Simsek, Arzu Poyanli, Ayşe Kubat Uzum, Fikret Buyukkaya, Emine Goknur Isik, Semen Onder, Nihat Aksakal, Zeynep Gozde Ozkan, Yasemin Sanli

**Affiliations:** 1 Department of Nuclear Medicine, Istanbul Faculty of Medicine, Istanbul University, Istanbul, Turkey; 2 Department of Radiology, Istanbul Faculty of Medicine, Istanbul University, Istanbul, Turkey; 3 Department of Internal Medicine, Division of Endocrinology and Metabolism, Istanbul Faculty of Medicine, Istanbul University, Istanbul, Turkey; 4 Department of Pathology, Istanbul Faculty of Medicine, Istanbul University, Istanbul, Turkey; 5 Department of General Surgery, Istanbul Faculty of Medicine, Istanbul University, Istanbul, Turkey

**Keywords:** Parathyroid adenoma, Primary hyperparathyroidism, ^68^Ga-Trivehexin, PET/CT, MIBI SPECT/CT

## Abstract

**Introduction:**

This study aims to assess ^68^Ga-Trivehexin PET/CT for detecting hyperfunctioning parathyroid tissue in comparison to [^99m^Tc]Tc-MIBI scintigraphy-SPECT/CT (MIBI scan) in patients with primary hyperparathyroidism (PHPT).

**Methods:**

The cohort comprised 13 patients diagnosed with PHPT based on biochemical analyses, including serum calcium, phosphorus, and parathyroid hormone (PTH) levels. Each participant underwent cervical ultrasonography, MIBI scan, and ^68^Ga-Trivehexin PET/CT imaging. Complementary 4D-CT and [^18^F]fluorocholine PET/CT were conducted in 7 patients. Ten lesions of 7 patients underwent PTH wash-out (WO) procedure. ^68^Ga-Trivehexin PET/CT findings were compared with other modalities and PTH-WO results.

**Results:**

Ten patients had sporadic PHPT, while 3 were diagnosed with MEN-1 syndrome-associated PHPT. One patient did not have any identifiable parathyroid lesion across the imaging modalities. On a patient-based analysis, MIBI scan and ^68^Ga-Trivehexin PET/CT identified parathyroid lesions in 10 and 11 patients, respectively. However, ^68^Ga-Trivehexin PET/CT detected 7 additional parathyroid lesions that were negative on the MIBI scan. Consequently, 17 lesions were identified and confirmed as hyperfunctioning parathyroid tissue through imaging, PTH-WO, or a combination of both modalities. In lesion-based evaluation, ^68^Ga-Trivehexin identified 16 lesions compared to 10 by MIBI scan, resulting in a detection rate of 94.1% and 58.8%, respectively. Notably, in three patients who underwent [^18^F]fluorocholine PET/CT, no lesions were detected; yet ^68^Ga-Trivehexin PET/CT successfully identified parathyroid lesions in two of these patients.

**Conclusion:**

Our study provides the first evidence that ^68^Ga-Trivehexin PET/CT can effectively identify hyperfunctioning parathyroid tissue with a high detection rate warranting further investigations to comprehensively explore its potential in PHPT management.

## Introduction

Primary hyperparathyroidism (PHPT) is one of the most encountered endocrine disorders, presenting with hypercalcemia and inappropriately normal to elevated parathyroid hormone (PTH) levels which is caused by hyperfunctioning parathyroid glands [1]. The leading cause of PHPT is adenomas that develop in one or more parathyroid glands [2]. Surgical intervention is the primary treatment for most patients necessitating accurate localization for guiding surgical interventions and improving patient outcomes [3]. [^99m^Tc]Tc-MIBI scintigraphy and SPECT/CT (MIBI scan) is the most used imaging modality alongside neck ultrasound (USG) in the conventional imaging work-up of hyperfunctioning parathyroid glands. However, its tendency to generate false-negative results, especially in cases of small or ectopic adenomas, poses a significant challenge [4, 5]. Additionally, interference from uptake due to concurrent conditions like thyroid nodules, thyroid cancer, and lymphadenopathy further complicates interpretation, particularly when suspected lesions are in close proximity to the thyroid gland [5]. These limitations prompt the exploration of novel methods. In the context of functional imaging, several studies have shown its usefulness highlighting the excellent diagnostic performance of [^18^F]fluorocholine PET/CT in similar difficult scenarios [6, 7]. Widespread use of this relatively new tracer has been restricted due to higher cost and limited availability in addition to licensing and reimbursement issues which can differ from one region to another.

^68^Ga-Trivehexin is a novel PET tracer targeting integrin receptors which are a family of cell surface receptors involved in cell-cell and cell-extracellular matrix interactions [8]. Among the integrin receptor subtypes, αvβ6 is implicated in various pathological processes, including tumor progression, making it a promising theranostic target for rapidly growing malignant cells [9–11]. In this regard, a key role of αvβ6 is to activate TGF-β by releasing it from its latent form bound to the latency-associated peptide. This activation subsequently triggers epithelial-mesenchymal transition, a process by which epithelial cells acquire mesenchymal characteristics, enhancing their migratory and invasive capabilities. Additionally, TGF-β activation inhibits cytotoxic T-cell activity, thereby weakening the immune response against the tumour and contributing to immune evasion [12, 13]. Consequently, αvβ6-expressing tumors such as pancreatic ductal adenocarcinoma and head and neck squamous cell carcinoma [14–16] have become prominent subjects of investigation for non-invasive imaging employing novel tracers like ^68^Ga-Trivehexin.

The incidental discovery of a parathyroid adenoma exhibiting notable tracer uptake on ^68^Ga-Trivehexin PET/CT prompted us to consider its potential in distinguishing parathyroid lesions. Subsequently, we aimed to investigate the performance of ^68^Ga-Trivehexin PET/CT for the localization of parathyroid adenomas in patients with PHPT in comparison to the MIBI scan.

## Methods

### Study design and patients

Thirteen patients diagnosed with biochemically proven PHPT underwent initial neck USG followed by conventional scintigraphic imaging. Subsequently, they underwent complementary ^68^Ga-Trivehexin PET/CT to pinpoint the localization of hyperfunctioning parathyroid tissue, which was retrospectively evaluated. Patients were referred for ^68^Ga-Trivehexin PET/CT if they met the following criteria: (1) patients with increased parathormone (PTH) levels, (2) no prior surgery in the neck area, (3) no medical history of neoplastic or inflammatory/infectious disease in the area of head and neck (4) no patients under 18 years old. This retrospective study was approved by the local institutional review board (Document no.2024/850) and written informed consent was obtained from all patients. The primary endpoint was to determine the detection rate of ^68^Ga-Trivehexin PET/CT in comparison to conventional scintigraphic imaging for localization of hyperfunctioning parathyroid tissue. The reference standard encompassed either biochemically confirmed parathyroid hyperfunction coupled with comprehensive neck imaging by USG and MIBI scan or USG-guided PTH concentration in the needle washout of fine-needle aspiration.

^**68**^**Ga-Trivehexin Synthesis**.

The synthesis of ^68^Ga-Trivehexin was conducted using an automated system (SCINTOMICS GmbH, Germany) under GMP standards. Single-use cassettes and reagent kits from ABX (Germany) ensured sterility and compliance. The Trivehexin precursor from Trimt GmBH (Germany) came in 50 μg vials for R&D. A ^68^Ge/^68^Ga generator (Eckert & Ziegler, IGG100, 1.85 GBq/50 mCi, Germany) was utilized. [^68^Ga]GaCl3 was eluted with 5 ml of 0.1 M HCl, purified via cation exchange, eluted with 1.5 ml of 5 M NaCl into a vial containing 50 μg Trivehexin in HEPES-buffer, heated for 15 min at 90 °C, passed through a C18 SepPak cartridge, and washed with 1 ml of water. The ^68^Ga-Trivehexin was eluted with 2 ml ethanol/water (50/50), filtered through a 0.22 μm sterile filter, and diluted with 0.9% NaCl to 15 ml into sterile vial. Radiochemical purity (> 95%) was confirmed by HPLC and TLC.

### ^68^Ga-Trivehexin imaging

Between 3 and 7 days after the MIBI scan, PET/CT acquisition was performed at 30 min post-intravenous injection of approximately 185 MBq of ^68^Ga-Trivehexin. An additional delayed imaging between 50 and 90 min was also performed to evaluate a potential washout, if any. Images were acquired for 3 min per bed position from the vertex to mid-thigh levels (Discovery IQ, GE Healthcare) and reconstructed using the Bayesian penalized likelihood reconstruction algorithm (Q.Clear, penalization factor of 300). All acquired data were transferred to the Advantage Workstation (version 4.7, GE Healthcare) for visual and quantitative evaluation.

### Conventional scintigraphic imaging

Dual-phase parathyroid scintigraphy was performed at 10 and 120 min after intravenous administration of 550 to 740 MBq [^99m^Tc]Tc-MIBI. Imaging was performed using a large field-of-view gamma camera (GE Discovery NM/CT 670) with low-energy, high-resolution collimators, including the entire neck and the chest down to the base of the heart in the field of view. At 60 min the planar imaging of the mediastinum followed by SPECT/CT imaging of the neck area was performed using acquisition parameters as follows; Matrix 128 × 128, zoom level 1.75, 60 projections, 30 s/frame, CT at 120 kV; 80 mAs, slice thickness 2 mm.

### Image interpretation and analysis

Two board-certified nuclear medicine physicians conducted a joint evaluation of the MIBI scan and ^68^Ga-Trivehexin PET/CT in consensus. The identification of positive findings for hyperfunctioning parathyroid glands encompassed all foci of uptake behind the thyroid lobes or in the upper mediastinum. For PET/CT images, a region of interest was drawn manually along the borders of the suspected lesions, and the maximum standardized uptake value (SUVmax) was calculated. All measurements were performed using the dedicated software of the manufacturer (GE Healthcare, AW 4.7). A positive lesion was characterized by increased activity within the suspected lesion surpassing that of the surrounding background. Hence, the quantity and specific sites of all parathyroid lesions were determined. Furthermore, an in-depth assessment was carried out to determine the presence and functional status of any thyroid nodules that may have been present. Laboratory screening results including serum calcium, phosphorus, and PTH alongside USG findings were also documented. All patients underwent USG, with an additional subset of three undergoing 4D-CT, and another three undergoing [^18^F]fluorocholine PET/CT scans for comprehensive evaluation. The description of SUVmax and laboratory results utilized median and range.

## Results

### Patient characteristics

The cohort consisted of 13 patients, (3 men and 10 women), with a mean age of 52.3 (range 28–73). Three patients had familial hyperparathyroidism associated with MEN-1 syndrome while 10 had sporadic disease. Key biochemical parameters conducted within one week of radiological imaging exhibited the following mean values within the study cohort: calcium (11 ± 0.79 mg/dL), PTH (136 ± 122.8 ng/l), albumin (4.6 ± 0.32 g/l), phosphorus (2.8 ± 0.44 mg/dL), vitamin D (19.3 ± 12.47 ng/dL), creatinine (0.8 ± 0.18 mg/dL), and creatinine clearance (95 ± 26.69 ml/min).

### Imaging studies preceding ^68^Ga-Trivehexin PET/CT

All patients underwent comprehensive neck evaluation using USG and MIBI scans, which involved employing SPECT/CT studies covering the anatomical region from the skull base to the heart base while employing dual-phase acquisition. Additionally, 4 patients underwent 4D-CT imaging of the neck, and 3 had [^18^F]fluorocholine PET/CT to complement the MIBI scan.

**Fig. 1:**
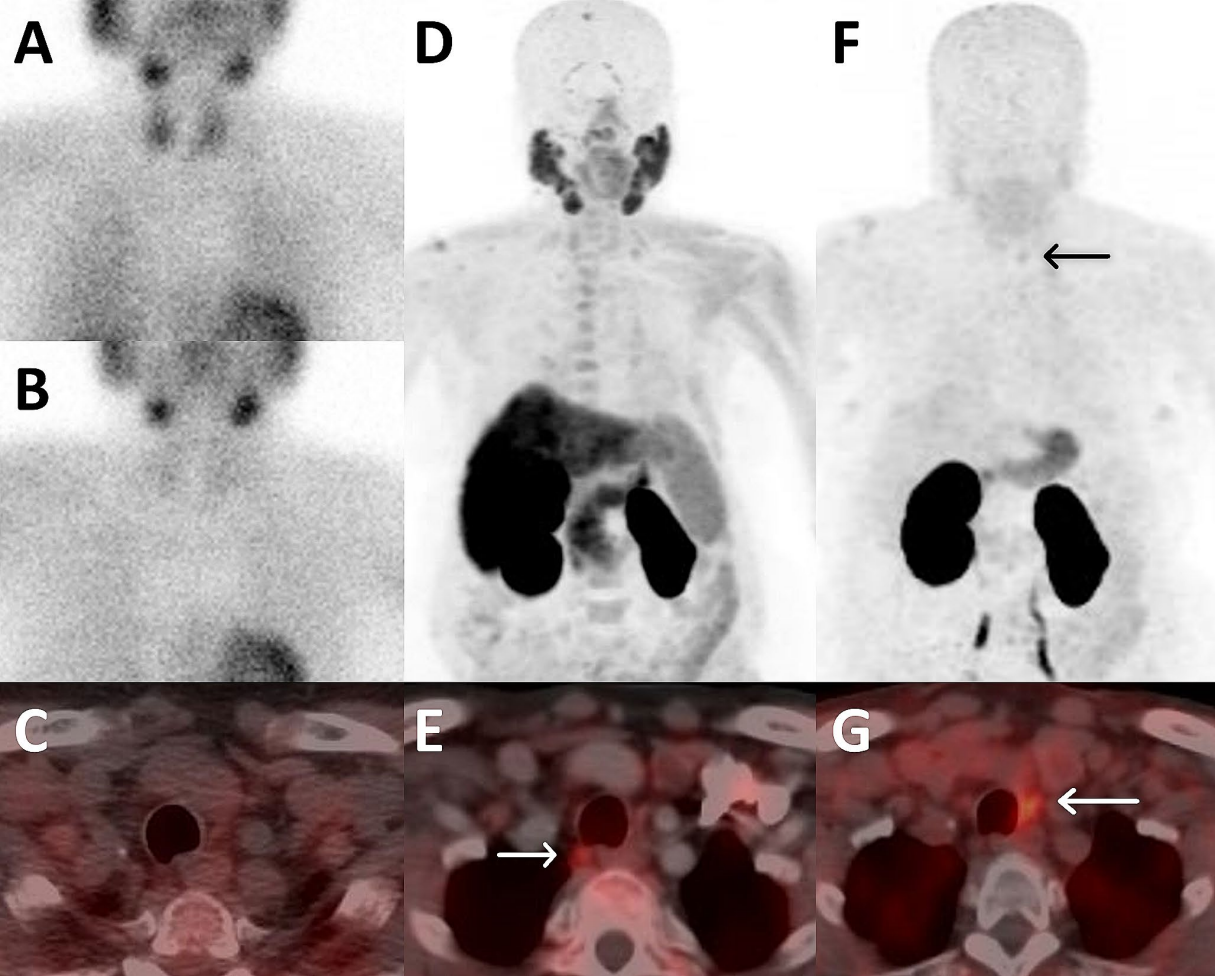
A 65-year-old woman with incidental PHPT (serum PTH: 108 ng/l Calcium: 11.5 mg/dL) underwent initial MIBI scan. However, both early and delayed planar (**A**, **B**) and SPECT/CT (**C**) images did not exhibit tracer uptake. Subsequent assessment with [^18^F]fluorocholine PET/CT (**D**) revealed uptake in an upper right paratracheal lesion (**E**). Additionally, ^68^Ga-Trivehexin PET/CT demonstrated uptake (**F**) in a lesion posterior to the left lower thyroid lobe on axial images (**G**). Further investigation through PTH-WO confirmed the false-positive uptake observed on [^18^F]fluorocholine PET/CT , while accurately identifying the uptake observed on ^68^Ga-Trivehexin PET/CT as indicative of a parathyroid lesion

**Fig. 2:**
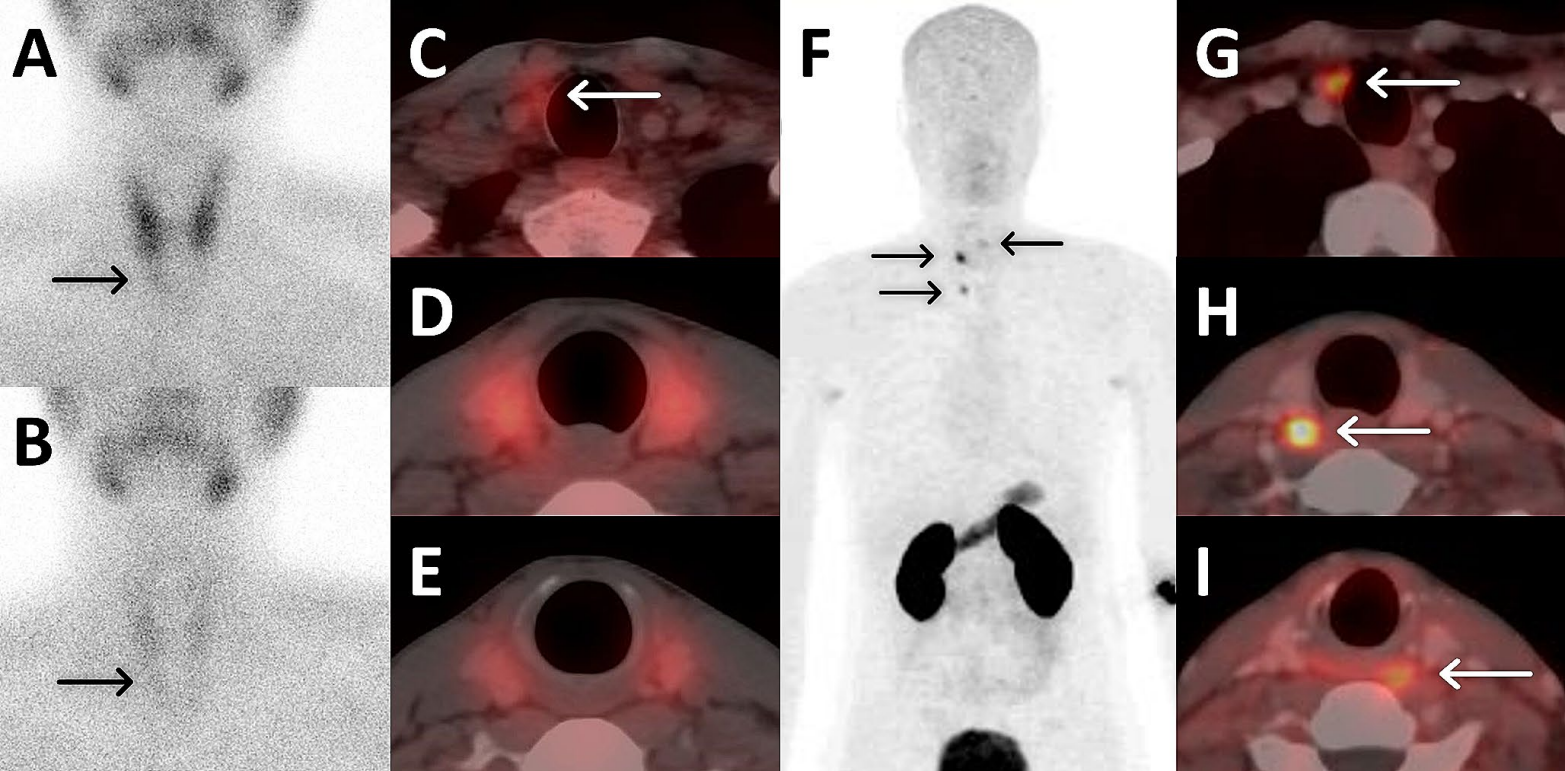
A 38-year-old man with MEN-1 syndrome and associated PHPT (PTH: 110 ng/l, Calcium: 11.4 mg/dL) underwent MIBI scan, revealing uptake in a lesion inferior to the right lobe of the thyroid gland on planar (**A**, **B**) and SPECT/CT (**C**) images. However, no accumulation was detected in lesions located posterior to the right lobe and in the left upper lobe (**D**, **E**). Subsequent ^68^Ga-Trivehexin PET/CT revealed uptake in all three lesions (**F**, **G**, **H**, **I**)

USG findings were negative for three patients. Among the remaining ten, USG revealed eight parathyroid lesions and identified five lesions with suspicious characteristics that were inconclusive to be determined as parathyroid. Subsequent 4D CT imaging was conducted on four patients; three of whom had negative USG (***Patients #4***,*** #9***,*** #6***) results. Similarly, 4D-CT could not identify any lesions in one USG-negative patient (***Patient #6***). However, in the other 2 USG-negative patients (***Patients #4***,*** #9***) it revealed 4 contrast-enhancing lesions 2 of which were reported as suspicious (Figs. 1 and 2). Consequently, our cohort encompassed a total of 17 lesions, with the mean size of the lesions’ largest dimension being 7.8 ± 4.5 millimeters.

Out of 17 suspected parathyroid lesions, 10 exhibited uptake on [^99m^Tc]Tc-MIBI SPECT/CT, indicating hyperfunctioning parathyroid adenomas. Consequently, the detection rate of the MIBI scan was determined to be 10 out of 17 for lesion-based analyses and 10 out of 13 for patient-based analyses. Notably, only one patient presented with multiple lesions on MIBI SPECT/CT. Additionally, the 3 patients who tested negative on the MIBI scan underwent [^18^F]fluorocholine PET/CT for further evaluation. However, [^18^F]fluorocholine PET/CT failed to detect any parathyroid lesions instead, introduced false positive uptake in the lymph nodes in one of the patients. The lymph nodes exhibiting uptake had been previously identified as suspicious; however, subsequent evaluation with PTH-WO confirmed their false positive [^18^F]fluorocholine status.

### ^68^Ga-Trivehexin PET/CT findings

In 11 out of 13 patients examined, ^68^Ga-Trivehexin PET/CT exhibited successfully identified parathyroid tissue. In one of the patients who tested negative with ^68^Ga-Trivehexin PET/CT, neither USG nor 4D-CT revealed a lesion (***Patient #6***). Furthermore, despite biochemical markers indicating otherwise, neither tracer uptake was detected at the parathyroid quadrants on the MIBI scan or [^18^F]fluorocholine PET/CT in this patient. In the other patient who tested negative with ^68^Ga-Trivehexin PET/CT, the MIBI scan showed mild uptake in the parathyroid lesion (***Patient #8***). Consequently, 11 patients tested positive for parathyroid adenomas by ^68^Ga-Trivehexin PET/CT achieving a detection rate of 84.1% in the patients-based approach. Excluding the patient with no suspicious lesion detected by any modality, a total of 17 lesions were assessed across 12 patients. Per lesion evaluation showed that ^68^Ga-Trivehexin PET/CT detected 16 out of 17 lesions achieving an impressive detection rate of 94.1%. The median SUVmax of the lesions was 5.99 (range 1.7 to 65.5) and median the lesion-to-background ratio was 4.5 (range 1.3 to 54.6). Table 1 presents an overview of patient characteristics, along with imaging and laboratory findings.

**Table 1:** Patients’ characteristics, imaging and laboratory findings

								Visual			Quantitative			
Patient		PTH (ng/l)	Ca (mmol/l)	Imaging	Lesion No	Dimensions (mm)	USG/4D-CT	MIBI Scan	^68^Ga-Trivehexin	Location	SUV_max_	LBR	PTH-WO	Key Findings
#1	Familial	122	10.6	USG	1	9 × 8	+	+++	+++	LULP	6.5	5	3068	*Significant ^68^Ga-Trivehexin uptake in the MIBI-negative lesion
					2	6 × 3	suspicious	+	+	LLLP	1.7	1.3	4999	
					3	5 × 3	suspicious	No uptake	+++	RLLP	5.3	4	2071	
#2	Sporadic	532	13.3	USG	4	22 × 17	+	+++	++	RLLP	4.5	3.5		*MIBI and ^68^Ga-Trivehexin agreement
#3	Sporadic	118	10.3	USG + 4D CT	5	9 × 3	+	No uptake	+++	RMLP	8.1	5.8		*Significant ^68^Ga-Trivehexin uptake *No MIBI uptake.
#4	Sporadic	108	11.5	USG + 4D CT	6	4 × 2	USG negative but CE on 4DCT	No uptake	+	LLLP	2.9	2.2	5000	*No MIBI or [^18^F]Fluorocholine uptake *The parathyroid lesion exclusively detected by ^68^Ga-Trivehexin PET/CT
#5	Sporadic	91	10.5	USG	7	6 × 3	+	+	+++	LMLP	7.3	5.6	5000	*Mild MIBI uptake versus intense ^68^Ga-Trivehexin activity.
#6	Sporadic	104	11.4	USG + 4D CT	Not Found	Not Found	USG and 4D-CT negative	No uptake	No uptake	Not Found	n/a	n/a	n/a	*False positive [^18^F]Fluorocholine uptake in the lymph nodes
#7	Familial	95	10.8	USG	8	8 × 5	+	+++	+++	LLLP	25.3	19.5	108	*Significant ^68^Ga-Trivehexin uptake in the MIBI-negative lesion
					9	5 × 3	suspicious	No uptake	+	RLLP	2.2	1.7	4292	
#8	Sporadic	79	10.6	USG	10	6 × 3	suspicious	+	No uptake	LMLP	-	-	170	*^68^Ga-Trivehexin missed the MIBI-positive lesion
#9	Familial	110	11.4	USG + 4D CT	11	6 × 4	USG negative; inconclusive CE 4D CT	No uptake	+++	RULP	11.7	9.8		*^68^Ga-Trivehexin PET/CT identified two additional lesions
					12	5 × 4	USG negative; CE on 4D CT	+	+++	RLLP	6.5	5.4		
					13	3 × 3	USG negative; inconclusive CE 4D CT	No uptake	+	LULP	4	3.3		
#10	Sporadic	179	11.3	USG	14	12 × 10	+	+++	+++	LLLP	65.5	54.6		*MIBI and ^68^Ga-Trivehexin agreement
#11	Sporadic	43	10.6	USG	15	6 × 3	suspicious	No uptake	+	RLLM intrathyroidal	4.5	3.7	707	*MIBI and [^18^F]Fluorocholine PET/CT missed intrathyroidal parathyroid lesion
#12	Sporadic	103	10.5	USG	16	8 × 3	+	++	++	LULP	4.4	3.7		*MIBI and ^68^Ga-Trivehexin agreement
#13	Sporadic	84	10.8	USG	17	13 × 6	+	+++	+++	LLLP	19.6	15		*MIBI and ^68^Ga-Trivehexin agreement

LULP: Left Upper Lobe Posterior; LLLP: Left Lower Lobe Posterior; RLLP: Right Lower Lobe Posterior; RMLP: Right Medial Lobe Posterior; LMLP: Left Medial Lobe Posterior; RULP: Right İupper Lobe Posterior; RLLM: Right Lower Lobe Medial; PTH: Parathormone; PTH WO: PTH Wash Out; LBR: Lesion to background ratio; n/a: not applicable

### Comparative findings

**Fig. 3:**
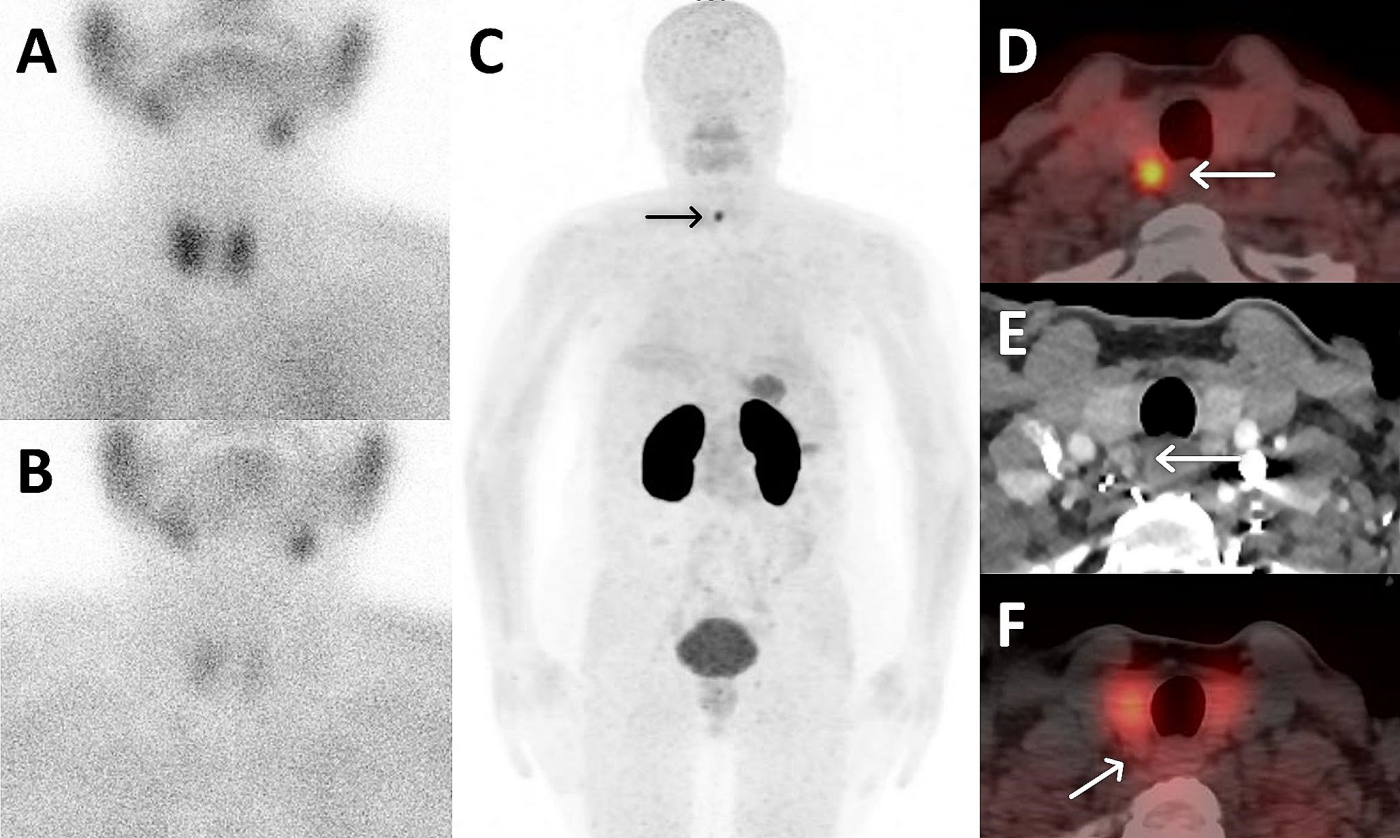
A 61-year-old woman diagnosed with PHPT (serum PTH: 118 ng/l Calcium: 10.3 mg/dL) underwent dual-phase MIBI scan showing no uptake (**A**, **B**) which prompted further investigation. Subsequent ^68^Ga-Trivehexin PET/CT displayed focal tracer activity, notably visible on maximum intensity projection images (**C**), located posterior to the left thyroid lobe on the axial plane (**D**). The lesion showing contrast enhancement (**E**) on 4D-CT exhibited no uptake on SPECT/CT images (**F**)

**Fig. 4:**
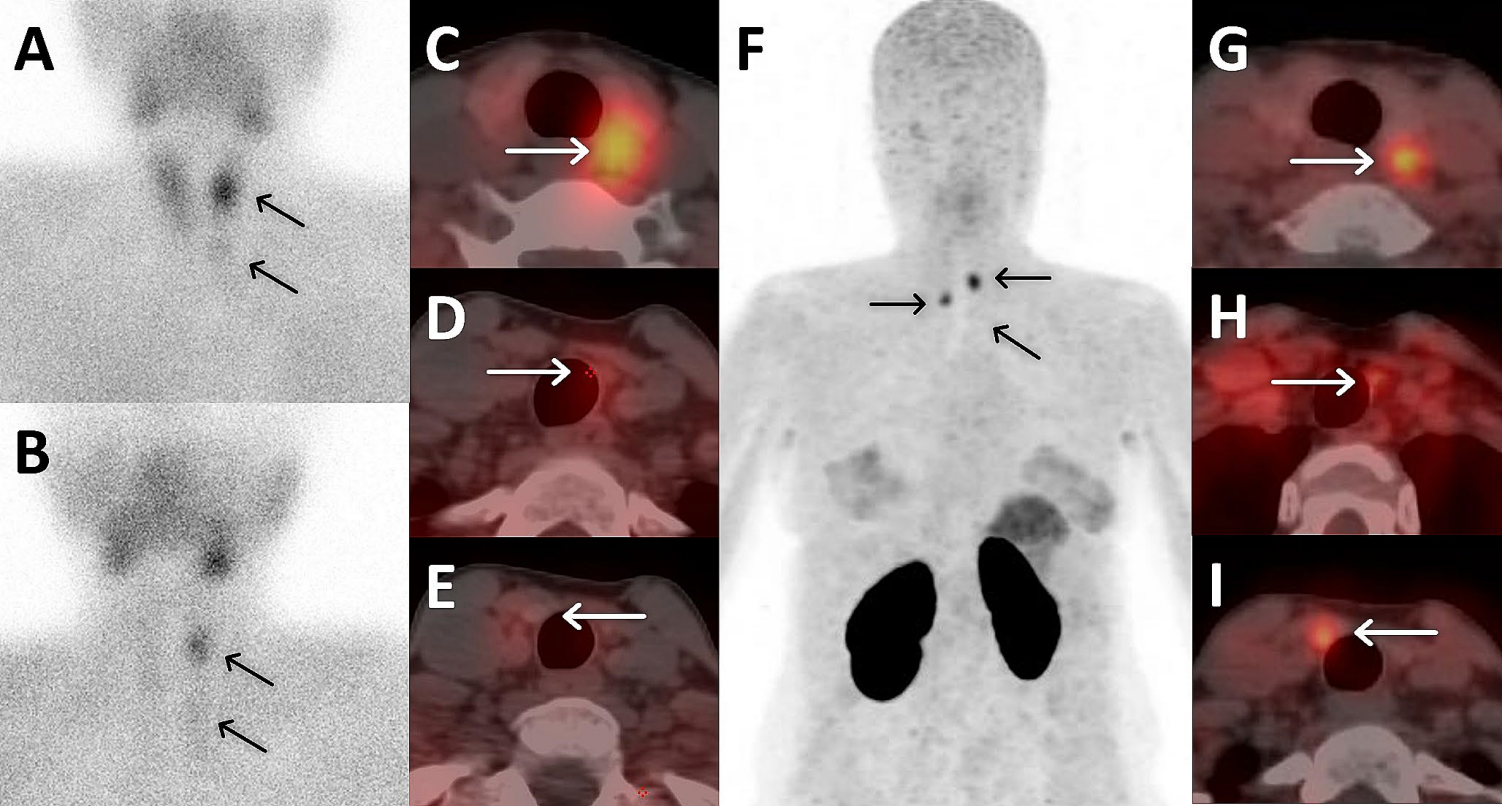
A 34-year-old woman diagnosed with MEN-1 syndrome and PHPT (PTH: 122 ng/l, Calcium: 10.6 mg/dL) underwent MIBI scan, showing intense activity in a lesion located posteriorly to the left lobe of the thyroid gland (**A**, **B**, **C**), and mild uptake in an inferiorly located lesion (**D**); yet no uptake was observed in a lesion inferior to the right lobe (**E**). Additional ^68^Ga-Trivehexin PET/CT uptake confirmed parathyroid pathology in all three lesions (**F**, **G**, **H**, **I**) through PTH wash-out

In one of the patients, neither a parathyroid lesion nor ectopic tissue was found. When comparing ^68^Ga-Trivehexin PET/CT to the MIBI scan on a per-patient basis, both modalities concurred in nine patients. In the subset of three cases where discordance was observed, ^68^Ga-Trivehexin PET/CT revealed parathyroid lesions in two patients with negative MIBI scans (Fig. 3) but failed to do so in one patient who tested positive on the MIBI scan. This was the sole parathyroid lesion out of 17, which did not show ^68^Ga-Trivehexin uptake in our cohort. However, ^68^Ga-Trivehexin PET/CT exclusively detected 7 lesions in 6 patients that were negative on the MIBI scan (Fig. 4). Of note, 2 of these patients had also undergone [^18^F]fluorocholine PET/CT which similarly yielded negative results. On a per-lesion basis, the MIBI scan revealed uptake in 10 lesions, while ^68^Ga-Trivehexin PET/CT demonstrated uptake in 16 lesions, translating to detection rates of 58.8% and 94.1%, respectively. Upon conducting a comparative analysis of nine lesions concurrently identified by both modalities, the lesions manifested varying degrees of tracer activity. Specifically, 4 lesions exhibiting mild MIBI uptake were clearly delineated by ^68^Ga-Trivehexin PET/CT whereas 5 lesions showed significant uptake on both scans.

### Other observations

^68^Ga-Trivehexin administration was well-tolerated, with no adverse effects. The biodistribution showed low soft tissue uptake in general. However, due to renal excretion and partial retention of the tracer, kidney activity was evident. One of the patients with MEN-1 syndrome had pituitary adenoma and multiple liver/bone metastases due pancreatic neuroendocrine tumor; however, none of the lesions besides parathyroid-related ones showed ^68^Ga-Trivehexin uptake. Moreover, a subset of patients demonstrated mild diffuse gastric uptake, while female patients demonstrated mild diffuse uterus activity. The mean SUVmax of gastric and uterine activity was 6.6 (range 3.6 to 9.3) and 6.3 (range 2.7 to 18.1) respectively. Additionally, one premenopausal patient exhibited diffuse ^68^Ga-Trivehexin uptake in the bilateral breast tissue (SUVmax: 3.4), while another patient displayed uptake within the fibrotic lung tissue (SUVmax: 4.4).

Thyroid gland evaluation revealed multiple millimetric nodules, predominantly cystic and benign as determined by USG. Thyroid scintigraphy did not identify any hypoactive nodules; however, 2 nodules from different patients (#5 and #12) exhibited uptake suggestive of hyperfunctioning. Patient #5, diagnosed with subclinical hyperthyroidism (normal T3 and T4 levels, low TSH: 0.06 mIU/L), demonstrated no ^68^Ga-Trivehexin activity in the thyroid nodule; in contrast to patient #12 (normal thyroid function) showing mild ^68^Ga-Trivehexin activity (SUVmax: 3.8). In our cohort an intrathyroidal nodule (Patient #11) was further evaluated and ultimately confirmed to be a parathyroid lesion. However, further evaluation of hyperactive nodule of patient #12 was not pursued for potential parathyroid origin despite the mild ^68^Ga-Trivehexin activity, as a parathyroid lesion had already been identified in this patient by PET/CT. Moreover, unlike patient#12, patient#11 was already under suspicion for a parathyroid lesion based on USG findings, which could not definitively determine whether the lesion was adjacent to the thyroid gland or located intrathyroidally. Furthermore, patient #11 despite having normal thyroid function and no thyroid nodules, showed heterogeneous mild ^68^Ga-Trivehexin uptake in the thyroid glands, which decreased significantly in the delayed images ( 90 min).

## Discussion

To the best of our knowledge, this study is the first to evaluate ^68^Ga-Trivehexin PET/CT in the parathyroid adenoma population and compare it to the MIBI scan. It shows that ^68^Ga-Trivehexin PET/CT exhibits significant tracer uptake across all but one parathyroid adenoma. Notably, comparative analysis unveiled the detection of 7 parathyroid lesions overlooked by the MIBI scan, while also providing a high lesion-to-background ratio and improved delineation of inconclusive lesions identified on the MIBI scan.

Parathyroid scintigraphy, using [^99m^Tc]Tc-MIBI is regarded as the primary radionuclide imaging for localization of hyperfunctioning parathyroid tissue in cases of PHPT. Dual-phase imaging, acquiring images at both early and delayed time points, has been found to be more accurate than single-phase acquisition. Moreover, comparative studies have demonstrated the superiority of SPECT/CT with [^99m^Tc]Tc-MIBI over planar or stand-alone SPECT imaging. A recent meta-analysis including 1236 patients reported a patient-based and lesion-based pooled detection rate of 88% for [^99m^Tc]Tc-MIBI SPECT/CT highlighting it as the standard method for detecting hyperfunctioning parathyroid glands. Similarly, EANM guidelines recommend utilizing at least one SPECT/CT study covering the anatomical region from the skull base to the heart base, while employing dual-phase acquisition [17]. This comprehensive approach maximizes the chances of precisely localizing hyperfunctioning parathyroid glands and helps appropriate clinical management and surgical planning for patients with pHPT. Considering this proven diagnostic accuracy, it was surprising that in our cohort 7 lesions out of 17 were negative on MIBI scan but positive on ^68^Ga-Trivehexin PET/CT, although these lesions were in the field of view of both dual-phase and SPECT/CT images. [^99m^Tc]Tc-MIBI SPECT/CT and dual-phase planar imaging have a reported combined sensitivity ranging from 87–97% [18]. However, several factors may affect the sensitivity of a MIBI scan. The size and location of the adenoma as well as the presence of concurrent thyroid pathologies or potential interference from high thyroid activity are reasons that merit consideration in this context. In this regard, in our cohort, multiple thyroid nodules of millimetric dimensions were noted. However, while two thyroid nodules showed uptake on ^99m^Technetium thyroid scintigraphy; and one of these nodules surprisingly demonstrated ^68^Ga-Trivehexin uptake on PET/CT, none of them showed uptake on the MIBI scan. Therefore, interfering thyroid nodules does not explain the negativity of the MIBI scan. In addition, it should be noted that except for one, thyroid nodules did not exhibit significant ^68^Ga-Trivehexin. On the other hand, in our cohort, comprising 17 detected lesions, it was observed that only three exceeded a diameter of 1 cm when assessed on 2D axial cross-sections. When examining lesions that were negative for MIBI but positive for ^68^Ga-Trivehexin, all seven lesions were of millimetric dimensions, with the largest measuring 9 × 3 mm. Moreover, of the 9 lesions that were positive for both [^99m^Tc]Tc-MIBI and ^68^Ga-Trivehexin, PET/CT provided superior delineation for 4 of these lesions. In this regard, the superiority and enhanced clarity of ^68^Ga-Trivehexin PET/CT can be attributed to the inherent characteristics of PET, particularly its superior spatial resolution compared to planar and [19] SPECT modalities.

MIBI scan using either dual planar or SPECT/CT or a combination of conventional methods is the imaging of choice in cases where USG fails to detect suspected adenoma. However, studies are also highlighting the suboptimal sensitivity of MIBI scans. For instance, in a study involving 658 patients, the detection rate of [^11^C]-methionine or [^11^C]-choline PET was notably higher compared to MIBI scans (79.4% vs. 25.4%). Notably, this study did not encompass the entirety of its patient cohort undergoing SPECT/CT, which warrants caution. Moreover, a recent meta-analysis has emphasized the superiority of [^99m^Tc]Tc-MIBI SPECT/CT over traditional planar imaging methods, demonstrating a pooled sensitivity of 86%. Considering these findings, it is essential to reassess the role of MIBI scans and explore alternative imaging modalities in the diagnosis of suspected parathyroid adenomas. In line with this context, EANM guidelines stated that it can be considered an alternative first-line imaging method. The utilization of [^18^F]fluorocholine PET/CT in the identification of hyperfunctioning parathyroid glands is presently acknowledged as an emerging area of interest [19]. Furthermore, several evidence-based data highlight the excellent diagnostic performance of this imaging method in detecting hyperfunctioning parathyroid glands with superior diagnostic performance surpassing all other currently accessible imaging modalities currently available. Notably, its reported sensitivity ranges from 90 to 97%, indicative of superior diagnostic performance [20–23]. Given the fact that not all patients underwent [^18^F]fluorocholine PET/CT in our cohort definitive conclusions regarding the diagnostic efficacy of ^68^Ga-Trivehexin PET/CT compared to [^18^F]fluorocholine PET/CT cannot be drawn. However, the impressive detection rate of 94.1% observed with ^68^Ga-Trivehexin PET/CT in our study cohort underscores its potential as a novel imaging tool. Given this promising rate which align with [^18^F]fluorocholine PET/CT related data in the literature, further studies comparing ^68^Ga-Trivehexin and [^18^F]fluorocholine PET/CT are essential to fully explore its clinical utility.

While our study demonstrates the promising diagnostic potential of ^68^Ga-Trivehexin PET/CT in localizing hyperfunctioning parathyroid adenomas, it is crucial to acknowledge that the unknown mechanisms driving integrin receptor overexpression in these adenomas represent the primary limitation of our investigation. Integrins are heterodimeric transmembrane glycoproteins, consisting of one α- and one β-subunit, and have a fundamental role in regulating crucial functions during cell adhesion, proliferation, migration/invasion, survival, and apoptosis. Many of the 24 human integrin subtypes known to date are involved in every step of cancer development which makes them compelling targets for further investigation and therapeutic intervention. In this regard, αvβ3 is a well-established integrin subtype for its role as a promoter of tumor angiogenesis whereas, the dimer αvβ6 targeted by ^68^Ga-Trivehexin is uniquely expressed by epithelial cells and plays a central role in the complex process of carcinogenesis. However, the mechanisms underlying the presence or overexpression of integrins in parathyroid adenomas remain unknown, posing a significant challenge.

The parathyroid glands are primarily composed of chief cells, responsible for PTH secretion, and oxyphil cells, distinguished by their high mitochondrial content. [^99m^Tc]Tc-MIBI diffuses passively through oxyphilic cell membrane and accumulates in the mitochondria enabling the use of MIBI scan to effectively detect parathyroid lesions. In our cohort, the reference standard for lesions was not based on post-surgical histopathological examination, which is a limitation of our study. Consequently, we cannot report the ratio of chief to oxyphilic cells, which could provide insight into MIBI-negative lesions detected by ^68^Ga-Trivehexin. The specific pathways underlying integrin overexpression in parathyroid adenomas remain elusive, presenting a significant barrier to fully exploiting the diagnostic implications of integrin receptor targeting in parathyroid imaging. In a previous research [24], it was found that PTH-related Protein (PTHrP) increases cell migration and invasion and upregulates the expression of the integrin α6β4. While PTH regulates serum calcium and phosphate levels, PTHrP, a hormone in the PTH family, has crucial developmental and physiological roles and is a primary cause of malignancy-related hypercalcemia. Additionally, PTH and PTHrP act through a common receptor [25], therefore it is plausible to suggest that PTH might also induce the overexpression of integrin receptors. However, ^68^Ga-Trivehexin binds specifically to the integrin receptor subtype αvβ6, which is known to be expressed in epithelial cells of the lung, skin, and kidney, but its expression in the parathyroid glands remains undetermined.

We also acknowledge noteworthy findings in our cohort that merit careful consideration, indicating potential variability in the specificity of this novel modality. For instance, a hyperfunctioning thyroid nodule on thyroid scintigraphy showed no ^68^Ga-Trivehexin or [^99m^Tc]Tc-MIBI uptake. However, a normal functioning thyroid nodule showing mild [^99m^Tc]NaTcO_4_ uptake surprisingly exhibited mild ^68^Ga-Trivehexin uptake. Another patient presented heterogeneous mild uptake in the thyroid glands which significantly decreased on the delayed images. ^68^Ga-Trivehexin uptake in these instances may cause an overlap in imaging characteristics between parathyroid and thyroid tissues underscoring the need for careful interpretation of ^68^Ga-Trivehexin PET/CT across different thyroid conditions. Nevertheless, the underlying mechanisms driving these uptake patterns remain unclear especially given the scarcity of data in the literature. Future studies should focus on these mechanisms, as understanding the molecular basis of integrin receptor expression could not only enhance the accuracy of imaging techniques but also pave the way for novel targeted therapeutic interventions in PHPT. Furthermore, we acknowledge limitations including the relatively small cohort size, potential patient selection bias, and the controlled study design, which preclude definitive sensitivity, specificity, and predictive value assessments.

## Conclusion

^68^Ga-Trivehexin demonstrates promising efficacy in detecting parathyroid adenomas compared to conventional imaging methods warranting further research as a potential new alternative tracer in the diagnostic work-up of hyperparathyroidism.

## Data Availability

The datasets generated during and/or analyzed during the current study are available from the corresponding author on reasonable request.
